# Differences in *Juniperus przewalskii* Rhizosphere Microbiomes across Age Classes: Community Diversity and Assembly

**DOI:** 10.3390/microorganisms11082094

**Published:** 2023-08-16

**Authors:** Qian Chen, Dengwu Li, Na Luo, Jinyan Yang

**Affiliations:** College of Forestry, Northwest A&F University, Xianyang 712100, China; chenqiansun1993@163.com (Q.C.); 18216581098@163.com (N.L.); yangjinyan710@163.com (J.Y.)

**Keywords:** age classes, co-occurrence network, community assembly, *Juniperus przewalskii*, rhizosphere

## Abstract

Evidence shows that biotic and abiotic factors have apparent diversity at different forest ages, leading to changes in rhizosphere microbiomes. However, the difference in diversity, co-occurrence pattern, and assembly of the rhizosphere microbial community among the different forest ages is still unclear. A total of 24 *Juniperus przewalskii* rhizosphere soil samples were selected from four representative age classes, using diameter at breast height (DBH) as a proxy for tree age (age class I: 5 < DBH ≤ 12.5 cm, age class II: 12.5 < DBH ≤ 22.5 cm, age class III: 22.5 < DBH ≤ 32.5 cm, and age class IV: DBH > 32.5 cm), and analyzed the structural characteristics of the soil microbial community by high-throughput amplicon sequencing. With the increase in age class, the microbial community α-diversity and β-diversity had an increased trend. The bacterial Shannon index in class II and class III were markedly higher than in class I. From class I to class IV, the relative abundances of dominant phyla such as Actinobacteria and Ascomycota decreased, and the relative abundances of Proteobacteria and Basidiomycota increased in contrast. The complexity and association stability of the bacteria and fungi community network structure increase with forest age. Stochastic processes mediated the assembly of soil bacterial communities, while deterministic processes played a more significant role in the assembly of fungal communities. In addition, the relative importance of deterministic components in the microbial community increased significantly with age class. Random forests suggested that soil pH, plant Shannon–Wiener index (H), and Pielou’s evenness index (J) were the most important driving factors of bacterial and fungal community assembly. Overall, these results provide information useful for understanding the generation and maintenance mechanisms of rhizosphere microbial communities across age classes.

## 1. Introduction

The root system can be likened to the gastrointestinal tract of a forest, where there is a significant exchange of nutrition and communication of signals [1,2,3]. The rhizosphere provides an excellent starting point for studying microbiome ecology [4]. There is evidence that biotic factors (e.g., plant diversity and development status of understory vegetation) [5,6,7] and abiotic factors, such as the accumulation of nutrient compounds [8,9,10] and the composition and decomposition of litter [11,12], have apparent diversity at different forest ages, which lead to changes in rhizosphere microbiomes [13,14]. Investigating the impact of forest age on soil microbial community diversity is an important area of research, and recent studies have reported varying effects on different microbial groups, with some showing no significant changes and others indicating significant increases or decreases [15,16]. Understanding the relationships between rhizosphere microbial communities and forest age and the basic ecological processes of different forest ages is crucial for the stability and sustainable development of forest ecosystems.

In soil ecosystems, microbial species coexist in complex arrays through positive (e.g., symbiosis) and negative (e.g., competition) associations [17,18], which can regulate the microbial community structure and thus adjust the functions it provides to the ecosystem [19,20]. Co-occurrence networks are increasingly used to infer linkages among microbial species in many different environments, including the human gut [21], oceans [22], and soils [23,24,25,26]. Networks analyses can be instrumental in describing network topological properties and inferring community assembly processes [26]. Therefore, network analyses have been considered a powerful approach in microbial ecology to provide meaningful information beyond the structural characteristics of the community [27,28]. For example, through network analysis Wang et al. [16] discovered that the complexity of bacterial networks increased during *Pinus tabulaeformis* plantation development, although there was no significant difference in the microbial community structure. However, little is known about the differences in the interspecific relationships of rhizosphere microbial communities across forest ages.

Understanding the mechanisms controlling community composition and diversity is a core topic of ecology, but little is known [29,30]. Niche theory and neutral theory constitute two important and complementary ecological processes that structure communities in many ecosystems [31,32]. The niche theory hypothesizes that deterministic processes govern community assembly dependent on species traits, interspecies relationships, and environmental conditions [33]. Conversely, the neutral theory asserts that stochastic processes are affected by birth, death, colonization, and extinction, emphasizing the role of drift and dispersal [34]. The challenge lies in quantifying their relative contribution in different forest ages and unraveling the factors mediating microbial community assembly [35,36,37]. Most current community assembly studies are limited to grassland and agricultural ecosystems [38,39]. The ecological processes underlying microbial community assembly in forest soils are still mysterious, particularly those relative contributions at different forest ages. This incomplete knowledge hinders effective forest conservation and management and may lead to forest ecosystem degradation.

*Juniperus przewalskii* Kom. is an endemic and dominant tree species widely distributed on the northeastern Qinghai–Tibet Plateau, playing a pivotal role in conserving water, mitigating regional drought, and maintaining soil stability [40,41]. This tree species thrives on the dry, infertile southern slopes at altitudes of 2600–4300 m, providing essential habitats for various understory vegetation and microbiome species [42]. We selected 24 *J. przewalskii* rhizosphere soil samples from four representative age classes, using diameter at breast height (DBH) as a proxy for tree age (age class I: 5 < DBH ≤ 12.5 cm, age class II: 12.5 < DBH ≤ 22.5 cm, age class III: 22.5 < DBH ≤ 32.5 cm, and age class IV: DBH > 32.5 cm). This study aimed to (1) analyze differences in the structure of rhizosphere microbial communities across age classes; (2) explore interspecies associations differences in rhizosphere microbial communities across age classes; and (3) understand the assembly processes variations in rhizosphere microbial communities across age classes.

## 2. Materials and Methods

### 2.1. Site Description and Rhizosphere Soil Sampling

This study was conducted in the Qilian Mountain Nature Reserve (97°25′–103°46′ E, 36°43′–39°36′ N), located on the northeast margin of the Qinghai–Tibet Plateau [43]. The reserve provides important ecological and biodiversity functions and is an ideal place for tree ecology research [44]. The annual average temperature ranges between 1 and 4 °C, and annual precipitation ranges between 144.4 and 389.9 mm [43].

Using diameter at breast height (DBH) as a proxy for tree age, 24 *J. przewalskii* rhizosphere soil samples were selected from four representative age classes (age class I: 5 < DBH ≤ 12.5 cm, age class II: 12.5 < DBH ≤ 22.5 cm, age class III: 22.5 < DBH ≤ 32.5 cm, and age class IV: DBH > 32.5 cm), after reviewing the literature and conducting field investigations [45,46]. Each plot was 20 m × 20 m. Detailed geographic information of each plot is shown in Table 1. The area of trees was 20 m × 20 m, with a shrub area of 5 m × 5 m, with a grassland area of 1 m × 1 m. In each plot, we measured the height and coverage of each species separately. Fine roots were collected from 6 randomly selected healthy trees from each plot. After shaking off the loose soil, we brushed off the closely adhered soil from each tree carefully and combined it into a rhizosphere soil sample. The samples were passed through a 2 mm sieve and transported with ice to the laboratory as soon as possible. All samples were divided into two parts: one portion was stored at −80 °C for microbial DNA analysis, and the other portion was air-dried for total nitrogen (TN), organic carbon (SOM), total phosphorus (TP), total potassium (TK), available nitrogen (AN), available phosphorus (AP), available potassium (AK), and pH analysis [47].

### 2.2. DNA Extraction and High-Throughput Amplicon Sequencing

Soil total DNA was extracted using the OMEGA Soil DNA Kit for soil (Omega Bio-Tek, Norcross, GA, USA) according to the manufacturer’s instructions. The V3-V4 hypervariable regions of the bacterial 16S rRNA gene were amplified with the primers 338F (5′-ACTCCTACGGGAGGCAGCA-3′) and 806R (5′-GGACTACHVGGGTWTCTAAT-3′). The ITS region of fungi was amplified using the ITS5F (5′-GGAAGTAAAAGTCGTAACAAGG-3′) and ITS2R (5′- GCTGCGTTCTTCATCGATGC-3′) primers. Sequencing was performed on the Illumina MiSeq platform at Personal Biotechnology Co., Ltd. (Shanghai, China). The raw sequencing data were processed using the Quantitative Insights into Microbial Ecology 2 (QIIME2, version 2019.4) software. Paired-end reads were merged and assigned to each sample by the unique barcodes. The high-quality sequences were clustered into amplicon sequence variants (ASVs) using the DADA2 pipeline in QIIME2.

### 2.3. Statistical Analysis

One-way variance analysis (ANOVA) was performed to compare the significance of soil chemical properties, community diversity, and taxonomic composition among different age classes using Tukey’s HSD test. Plant species richness (R), Shannon–Wiener diversity index (H), Simpson diversity index (D), and Pielou’s evenness index (J) were calculated following the equations described by Su and Shangguan [7]. The α-diversity was analyzed with the Picante package in R (4.0.2). Principal-coordinate analysis (PCoA) and permutational multivariate analysis of variance (PERMANOVA) analyses were conducted to visualize and digitize β-diversity differences based on Bray–Curtis distances. ASVs with relative abundance above 0.05% were chosen to establish a co-occurrence network. Based on Spearman’s correlation coefficient *r* > 0.8 and *p* < 0.01, a robust correlation was constructed [48]. A set of topological features was calculated using Gephi-0.9.6 software, and networks were visualized [49]. The null model based on the normalized stochasticity ratio (NST) was calculated to estimate the relative importance of stochastic and deterministic processes using the NST package in R (4.0.2). The neutral model developed by Sloan et al. [50] was used to assess the contribution of stochastic processes in community assembly. Biotic and abiotic factors were standardized; then, the correlation between these factors and microbial community diversity was evaluated using Spearman’s correlation analysis. The Mantel test and Spearman’s correlation analysis were conducted to analyze the driving forces affecting microbial community composition at the phylum level, using the linkET package in R (4.0.2). Random forests were performed to determine the role of these factors in microbial community assembly.

## 3. Results

### 3.1. Vegetation and Soil Chemical Characteristics

The plant diversity index showed significant differences among the different age classes (*p* < 0.05) and reached their highest values in class III with the increase in age class (Table 2). In addition, most of the measured soil characteristics also had significant differences in nutrient contents and pH values among the different age classes (*p* < 0.05) (Table 2). Soil pH was slightly alkaline, and the pH was between 7.62 and 8.19. The contents of TN, SOM, AN, and AP in class I were significantly lower than those in class IV. The soil TP value was the lowest in class II, and the soil AK value was the highest in class III relative to the other age class.

### 3.2. Microbial Community Diversity and Composition

**Figure 1:**
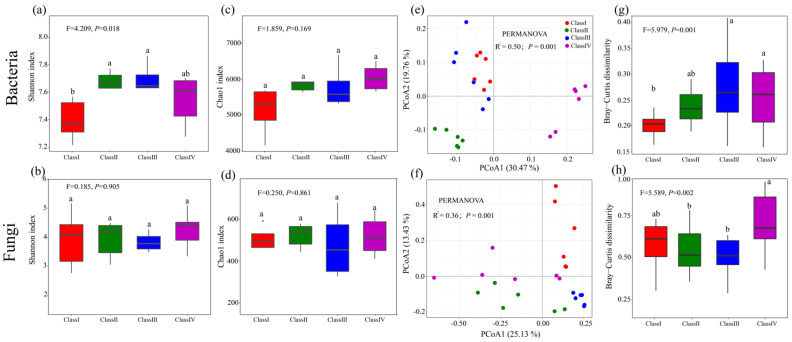
Diversity of bacterial and fungal communities in *J. przewalskii* under different age classes. (**a**–**d**) Boxplot displayed the differences in the Shannon index and Chao1 index of bacterial and fungal communities under different age classes. (**e**,**f**) Principal-coordinate analysis (PCoA) of bacterial and fungal communities based on Bray–Curtis distances. (**g**,**h**) The pairwise Bray–Curtis dissimilarity of bacterial and fungal communities under different age classes. Different letters above columns represent significant differences (*p* < 0.05) among groups according to the least significant difference (LSD) test.

A total of 2,061,534 and 2,578,202 high-quality sequences from the 24 rhizosphere samples were obtained by Illumina MiSeq sequencing of the bacterial 16S rRNA gene and fungal ITS gene, which were separated into 63,103 and 6283 ASVs, respectively. With the increase in age class, the microbial community α-diversity (Shannon and Chao1 index) had increased trends (Figure 1). The rhizosphere soil had a greater bacterial Shannon index in class II and class III compared with a lower bacterial index in class I (*p* < 0.05) (Figure 1a). The bacterial Chao1 index and fungal α-diversity were not significantly different among the four age classes (Figure 1b–d). PCoA analyses showed that soil bacterial and fungal samples from the same age class clustered together in many cases, confirmed by PERMANOVA analyses (R^2^ = 0.50, *p* = 0.001 and R^2^ = 0.36, *p* = 0.001, respectively) (Figure 1e,f). With the increase in age class, the microbial community β-diversity had increased trends (Figure 1g,h). The bacterial communities in class III and class IV displayed significantly higher β-diversity than those in class I (*p* < 0.01) (Figure 1g). The fungal communities in class IV displayed significantly higher β-diversity than those in class II and III (Figure 1).

**Figure 2:**
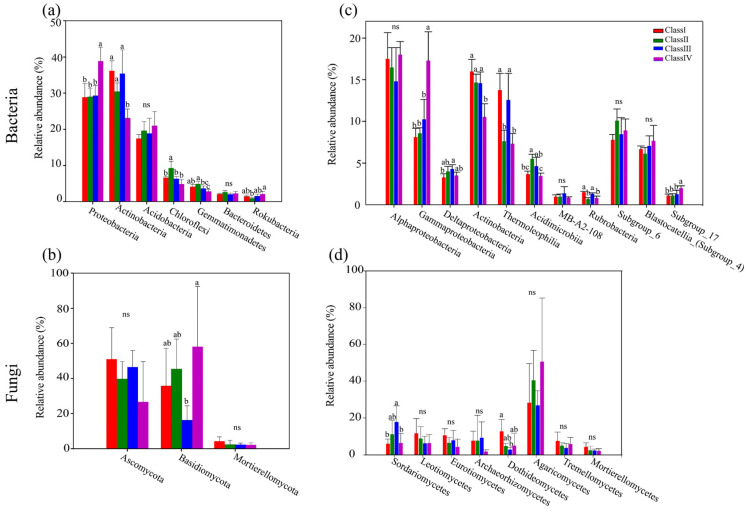
Composition of bacterial and fungal communities in *J. przewalskii* under different age classes. (**a**,**b**) The relative abundance (%) of dominant bacterial taxa and fungal taxa at the phylum level. (**c**,**d**) The relative abundance (%) of dominant bacterial taxa and fungal taxa at the class level. Different letters above columns represent significant differences (*p* < 0.05) among groups according to the least significant difference (LSD) test, ns: not significant.

For bacteria, the relative abundances of seven phyla exceed 1%, including Proteobacteria, Actinobacteria, Acidobacteria, Chloroflexi, Gemmatimonadetes, Bacteroidetes, and Rokubacteria (Figure 2a). There were significant differences in the relative abundances of those phyla among the different age classes (Figure 2a). From class I to class IV, the relative abundance of Proteobacteria (from 28.92% to 38.84%) was increased (*p* < 0.001), and the relative abundance of Actinobacteria (from 36.18% to 23.06%) was decreased in contrast (*p* < 0.001) (Figure 2a). Specifically, the relative abundance of Gammaproteobacteria within the phylum Proteobacteria in class IV increased significantly by 9.16% relative to class I (*p* < 0.001), while the relative abundances of classes such as Actinobacteria and Thermoleophilia within the phylum Actinobacteria in class IV decreased significantly by 5.44% and 6.43% relative to the class I, respectively, (*p* < 0.001) (Figure 2c). For fungi, the relative abundances of three phyla exceed 1%, including Ascomycota, Basidiomycota, and Mortierellomycota (Figure 2b). From class I to class IV, the relative abundance of Ascomycota (from 51.11% to 26.65%) was decreased (*p* > 0.05), and the relative abundance of Basidiomycota (from 35.83% to 58.08%) was increased in contrast (*p* < 0.05) (Figure 2b). Of them, the relative abundance of classes such as Sordariomycetes and Dothideomycetes within the phylum Ascomycota had significant differences among the four age classes but showed the reverse trend (*p* < 0.05) (Figure 2d).

### 3.3. Microbial Co-Occurrence Patterns

**Figure 3:**
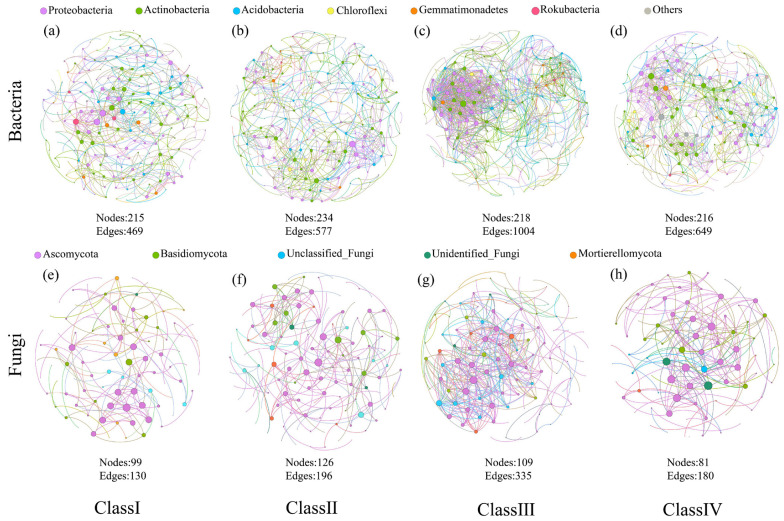
Network analyses showing the co-occurrence patterns of bacterial (**a**–**d**) and fungal (**e**–**h**) communities at the ASV level in *J. przewalskii* under different age classes.

Co-occurrence networks and multiple topological features were used to visualize and digitize the potential biotic associations of *J. przewalskii* rhizosphere microbiomes in different age classes (Table 3 and Figure 3). The bacterial and fungal networks of class III contained the greatest number of edges (1004 and 335, respectively) and average degree (4.61 and 3.07, respectively), whereas the bacterial and fungal networks of class I comprised the lowest number of edges (469 and 130, respectively) and average degree (2.18 and 1.31, respectively) (Table 3). Each network had a high modularity value > 0.4, indicating that their modular structures were not randomly constructed (Table 3). For bacterial networks, more than half of the correlations were positive, and the network nodes primarily belonged to Proteobacteria (37.16–39.81%), Actinobacteria (27.78–31.20%), and Acidobacteria (17.13–18.35%) (Figure 3a–d). The fungal networks showed more positive correlations than bacterial networks, and the network nodes were mainly affiliated with Ascomycota (55.96–65.60%) and Basidiomycota (11.20–18.52%) (Figure 3e–h).

### 3.4. Quantifying Community Assembly Processes

**Figure 4:**
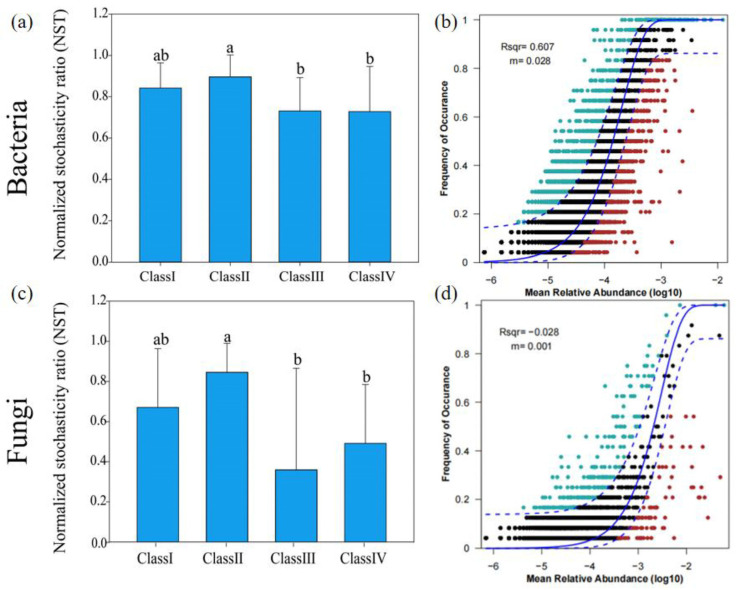
Niche-based and neutral-based processes in the assembly of the *J. przewalskii* rhizosphere microbial community under different age classes. Null models assessing the relative importance of stochastic and deterministic processes in the bacterial (**a**) and fungal (**c**) community assembly, with an NST greater than 0.5 indicating the dominance of stochastic processes, and, conversely, an NST of less than 0.5 indicating the dominance of deterministic processes. Different letters above columns represent significant differences (*p* < 0.05) among groups according to the least significant difference (LSD) test. Neutral models evaluated the role of stochastic processes in bacterial (**b**) and fungal (**d**) community assembly. The solid blue line is the best fit to the neutral community model, and the dashed blue line indicates 95% confidence intervals around neutral community model prediction. ASVs that occur more or less frequently than predicted by the model are shown in green and red, respectively. R^2^ represents the goodness of fit to the model, and m represents the species migration rate.

Normalized stochastic ratio (NST) and neutral model were used to analyze the role of stochastic and deterministic processes of *J. przewalskii* rhizosphere microbiomes in different age classes. The assembly of the rhizosphere soil bacterial communities was dominated by stochastic processes (NST > 0.5) (Figure 4a). In contrast, deterministic selections had a greater effect on rhizosphere fungal communities (NST < 0.5) (Figure 4c). In addition, the bacterial community assembly also fitted the neutral model well (R^2^ = 0.607, m = 0.028) (Figure 4b). The fungal community assembly did not fit the neutral model well (R^2^ = −0.028, m = 0.001) (Figure 4d). As the age class increases, the NST values of bacterial and fungal communities significantly decrease (*p* < 0.05) (Figure 4a,c), indicating that the relative importance of deterministic processes in rhizosphere bacterial and fungal communities increased with forest age. The average niche breadth width in the bacterial community (2.76) was larger than that in the fungal community (1.37).

### 3.5. Factors Influencing the Microbial Community

**Figure 5:**
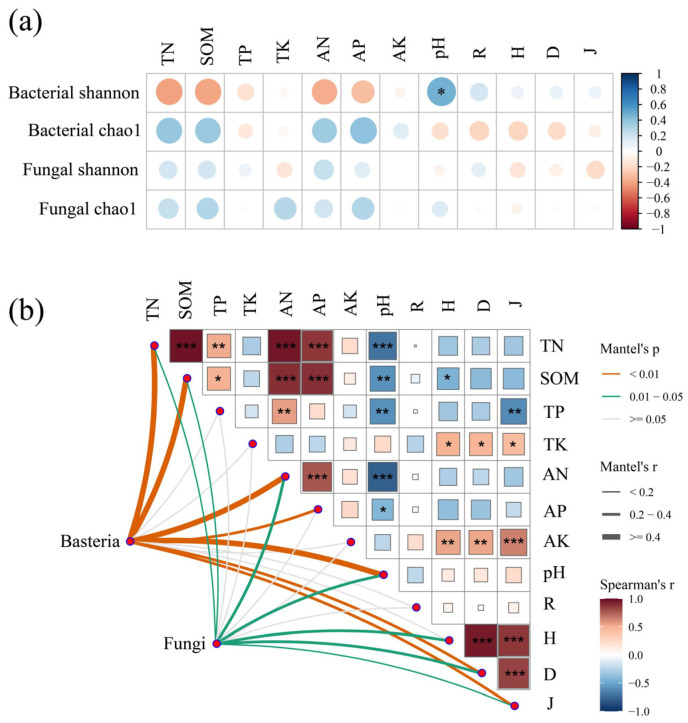
(**a**) Spearman’s correlation heat map of microbial community diversity with abiotic and biotic factors. (**b**) Correlation analysis between abiotic and biotic factors and the microbial community. Pairwise comparisons between factors are shown in a color gradient, with individual boxes containing Spearman’s correlation coefficient and indication of the level of significance: * *p* < 0.05, ** *p* < 0.01, *** *p* < 0.001. Mantel tests for the correlations between factors and microbial community composition at the dominant phylum levels (Spearman’s correlations, permutations = 9999).

**Figure 6:**
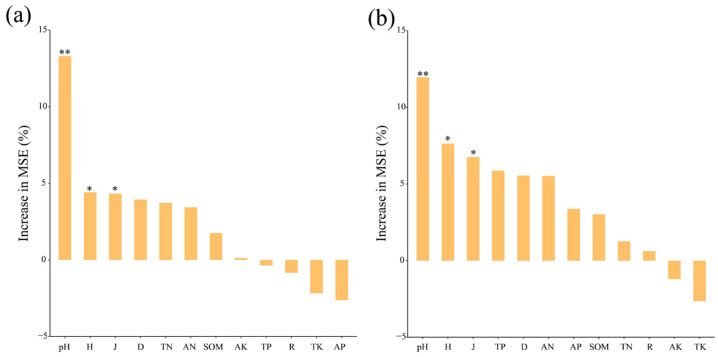
Random forests determined the role of plant diversity and soil properties in the bacterial (**a**) and fungal (**b**) communities’ assembly. Abbreviations: R: Plant species richness; H: Shannon–Wiener index; D: Simpson diversity index; J: Pielou’s evenness index; TN, total nitrogen; SOM; soil organic carbon; TP, total phosphorus; TK, total potassium; AN, available nitrogen; AP, available phosphorus; AK, available potassium. Asterisks indicate significant differences (*, *p* < 0.05, **, *p* < 0.01).

According to the correlation heat map, the correlation coefficients between microbial α-diversity (Shannon and Chao1 index) and these plant diversity and soil factors were small (*r* < 0.4). Only pH was significantly positively correlated with the bacterial Shannon index (*p* < 0.05) (Figure 5a). In addition, the Mantel test showed that the composition of dominant phyla in the bacterial community was significantly correlated with TN, SOM, AN, AP, pH, D, and J (*p* < 0.01), and the composition of dominant phyla in the fungal community was significantly correlated with TN, SOM, AN, pH, H, D, and J (*p* < 0.05) (Figure 5b). Random forests showed that pH, H, and J were the key predictors of soil bacterial and fungal community assembly (Figure 6).

## 4. Discussion

### 4.1. Microbial Community Diversity and Composition

Rhizosphere microbiomes play a critical role in aboveground vegetation and soil health, quality, function, and ecological sustainability [36]. With the increase in age classes, the rhizosphere microbial community α-diversity had an increasing trend (Figure 1a–d), which had been corroborated in previous studies [51,52]. For example, the diversity level of rubber tree plantations is highest at the ages of 10, 13, and 18-years-old, and lowest at the age of 5 years old [53]. Wan et al. [15] have revealed that *Ormosia hosiei* soil microbial community diversity was significantly affected by stand age. The reason for this might be that there were relatively high biomass, plant diversity, and nutrient contents at the old tree sites, which are significantly different from those at the young tree sites (Table 2) [54,55].

Proteobacteria, Actinobacteria, and Acidobacteria were the dominant bacterial phyla (Figure 2a) and Ascomycota and Basidiomycota were the dominant fungal phyla (Figure 2b), which are generally consistent with other forest soils [56,57,58]. In this study, there were significantly different bacterial and fungal community compositions in different age classes (Figure 2a,b). The histogram showed that higher relative abundances of Actinobacteria and Ascomycota were observed in class I, while higher relative abundances of Proteobacteria and Basidiomycota were observed in class IV (Figure 2a,b). The significant differences in the dominant phyla taxa among the four age classes may be explained by different life history strategies [59]. Proteobacteria and Basidiomycota have high nutritional requirements and are defined as copiotrophic taxa [60,61], and their relative abundance increases with an increase in resources [15,16], whereas Actinobacteria and Ascomycota belong to the oligotrophic taxa and are suitable for growth in a stressful environment [62].

The results showed that pH was the factor affecting microbial community diversity and composition (Figure 5), which is in general agreement with the previous studies [63]. Soil pH is the environmental factor that plays the most significant role in microbial community structure [5,6,7]. In addition, Mantel tests showed that H, D, J, TN, SOM, and AN were the main forces affecting microbial community composition (Figure 5b). Soil nitrogen (N) and carbon (C) are important nutrients for bacterial and fungal growth and activity [8]. Soil bacterial richness was positively correlated with plant species diversity [54,55].

### 4.2. Microbial Co-Occurrence Patterns

In soil ecosystems, microbial species coexist in arrays to form complex interspecies relationships, which are important factors affecting microbial community biodiversity [64]. Network analysis has been used to help reveal complex interspecies associations within microbial communities at different environmental changes [26]. In a co-occurrence network analysis, the greater number of edges and higher average degree, graph density, clustering coefficient, and average path length indicated higher connectivity and more complex interspecies relationships of microbial communities [65]. In this study, microbial communities in class III showed higher connectivity and complexity than those in class I and class II, indicating that the complexity of the microbial networks tended to increase with forest age (Table 3 and Figure 3), which led to higher community stability [66]. This finding may be attributable to the increase in plant diversity and nutrient content availability throughout their growth cycle [23]. Wang et al. [16] have revealed that the complexity of the *Pinus tabulaeformis* bacterial networks tended to increase markedly from 15 years to 60 years [16]. Similarly, bacterial species formed more complex and stable network structures under 20 and 30 years than 10-year-old pomelo trees [10].

Positive links could be attributed to cooperation, while negative relationships may be caused by competition [27]. In our work, co-occurrence patterns showed relatively high positive correlations between nodes regardless of age class (Table 3), implying that microorganisms adapt to similar ecological niches through interspecific cooperation [1]. The primary network nodes belonged to Proteobacteria, Actinobacteria, Acidobacteria, Ascomycota, and Basidiomycota (Figure 3a,b), further suggesting that Proteobacteria, Actinobacteria, and Acidobacteria were the most abundant bacterial phyla, and Ascomycota and Basidiomycota were the most abundant fungal phyla. These dominant phyla taxa play vital roles in maintaining the structure and function of soil ecosystems [17,18].

### 4.3. Microbial Assembly Processes

The assembly of microbial communities is controlled by stochastic processes (neutral theory) and deterministic processes (niche theory) based on the four elementary ecological processes: drift, speciation, dispersal, and selection [67]. Calculating and weighing the mechanisms that generate and maintain biodiversity is an important topic in soil ecosystems [31]. In the present study, the null model and neutral model show that bacterial community assembly was dominated by stochastic processes, while deterministic processes have taken a major role in the fungal community assembly processes (Figure 4). These results suggest that bacterial communities are less sensitive to changes in biotic and abiotic factors compared to fungal communities [68,69]. Similarly, Ku et al. [24] found that stochastic processes mediated the assembly of the rhizosphere bacterial communities, while deterministic processes mediated the assembly of the rhizosphere fungal communities of *Robinia pseudoacacia*. One possible explanation is that the increased plant diversity and accumulation of nutrient compounds with increasing forest age widened the habitat niche breadth of the rhizosphere microbial community and thus weakened environmental filtering [16]. In this study, there was a larger niche breadth width in the bacterial community than that in the fungal community. Chen et al. [31] reported that organisms with wider niche breadths might have greater metabolic plasticity, indicating that stochastic processes had a bigger influence on community assembly in the bacterial community compared with the fungal community. The other possible explanation is the size-plasticity hypothesis (body size effect) that smaller organisms (bacteria) are less environment filtered than larger organisms (fungi) [68,69,70]. Furthermore, with the increase in age class, the relative influence of deterministic processes in rhizosphere bacterial and fungal communities increased significantly (Figure 4a,b), suggesting that the relative importance of stochasticity declined, and that of deterministic selection increased as forest development stages increased [71]. This finding may be attributable to the increase in rhizosphere microbial community β-diversity (Figure 1g,h). Deterministic processes emphasize the role of environmental filtering, which lead to large changes in community composition (higher β-diversity) when the habitat conditions are different [72]. These changes may be due to the complexity of species characteristics or biological associations. In deterministic processes, not only environmental conditions but also biological relationships have a great influence on community assembly [73].

The random forests showed that assembly mechanisms of microbial communities were mediated by pH, H, and J. Soil pH was the most strong predictor of microbial community assembly (Figure 6). Tripathi et al. [32] reported that pH mediates the balance between stochastic and deterministic assembly of bacteria. Yang et al. [74] found that pH was most strongly correlated with soil microbial community structural characteristics and assembly mechanisms. Our study also demonstrated that plant diversity also played a vital role in microbial community assembly.

## 5. Conclusions

Our study provided compelling evidence that different forest ages affect the composition, structures, interspecies relationships, and assembly processes of the *J. przewalskii* rhizosphere microbial community. With the increase in age class, the microbial community α-diversity and β-diversity had an increased trend. The complexity and association stability of the network structure of bacteria and fungi increase with forest age. The bacterial community assembly was dominated by stochastic components, while the fungal community assembly was dominated by deterministic components. The relative importance of deterministic processes increased with forest age. Soil pH and plant diversity played a vital role in microbial community assembly.

## Figures and Tables

**Table 1:** Detailed distribution of the sampling sites of *J. przewalskii* forest under different age classes.

Age Class	Average Diameter (cm)	Longitude (° E)	Latitude (° N)	Altitude (m)
Class I	10	102.4325	37.1336	2730
Class II	18.5	102.4344	37.1338	2770
Class III	25.2	102.1519	37.1425	2700
Class IV	35.4	102.4611	37.1325	2820

**Table 2:** Plant diversity and soil chemical properties of *J. przewalskii* forest under different age classes.

Properties	Class I	Class II	Class III	Class IV
Species richness	11.25 ± 1.25 ^a^	10.83 ± 0.85 ^a^	13.17 ± 2.49 ^a^	11.25 ± 0.75 ^a^
Shannon–Weiner index	1.36 ± 0.16 ^b^	1.45 ± 0.05 ^b^	1.95 ± 0.23 ^a^	1.21 ± 0.17 ^b^
Simpson diversity index	0.56 ± 0.14 ^b^	0.64 ± 0.03 ^ab^	0.76 ± 0.07 ^a^	0.44 ± 0.07 ^b^
Pielou’s evenness index	0.53 ± 0.15 ^bc^	0.75 ± 0.02 ^ab^	0.88 ± 0.05 ^a^	0.45 ± 0.15 ^c^
TN (g/kg)	2.47 ± 0.50 ^b^	2.48 ± 0.92 ^b^	2.69 ± 0.68 ^b^	7.35 ± 1.05 ^a^
SOM (g/kg)	54.07 ± 14.01 ^b^	71.68 ± 23.12 ^b^	57.82 ± 16.54 ^b^	112.83 ± 21.30 ^a^
TP (g/kg)	0.73 ± 0.07 ^a^	0.49 ± 0.12 ^b^	0.63 ± 0.02 ^a^	0.74 ± 0.05 ^a^
TK (g/kg)	20.59 ± 3.66 ^a^	18.97 ± 1.11 ^a^	18.80 ± 1.08 ^a^	17.54 ± 0.86 ^a^
AN (mg/kg)	69.95 ± 14.19 ^b^	62.84 ± 15.33 ^b^	75.71 ± 17.63 ^b^	159.94 ± 13.92 ^a^
AP (mg/kg)	2.74 ± 0.61 ^b^	4.19 ± 1.20 ^ab^	3.31 ± 1.28 ^b^	5.39 ± 0.58 ^a^
AK (mg/kg)	94.83 ± 20.93 ^b^	143.17 ± 34.65 ^b^	205.75 ± 39.24 ^a^	130.17 ± 16.55 ^b^
pH	8.04 ± 0.05 ^ab^	8.19 ± 0.10 ^a^	7.89 ± 0.04 ^b^	7.62 ± 0.12 ^c^

Different letters above columns represent significant differences (*p* < 0.05) among groups according to the least significant difference (LSD) test. Abbreviations: TN, total nitrogen; SOM, soil organic carbon; TP, total phosphorus; TK, total potassium; AN, available nitrogen; AP, available phosphorus; AK, available potassium.

**Table 3:** Topological features of the *J. przewalskii* rhizosphere microbial community under different age classes.

Topological Properties	Bacteria				Fungi			
	Class I	Class II	Class III	Class IV	Class I	Class II	Class III	Class IV
Nodes	215	234	218	216	99	126	109	81
Edges	469	577	1004	649	130	196	335	180
Average degree	2.18	2.47	4.61	3.01	1.31	1.56	3.07	2.22
Average path length	2.28	2.26	2.55	2.42	1.47	1.44	2.16	1.71
Network diameter	9	8	9	7	4	4	6	5
Graph density	0.01	0.01	0.02	0.01	0.01	0.01	0.03	0.03
Average clusteringcoefficient	0.07	0.06	0.09	0.11	0.06	0.13	0.05	0.09
Modularity	0.76	0.73	0.58	0.68	0.82	0.86	0.54	0.64
Positive edges (%)	53.09	53.20	52.39	53.06	56.15	68.37	67.76	88.33
Negative edges (%)	46.91	46.80	47.61	46.94	43.85	31.63	32.24	11.67

## Data Availability

The raw reads of all soil samples in this study were deposited in the SRA of NCBI database under bacteria accession numbers PRJNA912361 and fungi accession numbers PRJNA912260.
